# Population Pharmacokinetics of an Indian F(ab')_2_ Snake Antivenom in Patients with Russell's Viper (*Daboia russelii*) Bites

**DOI:** 10.1371/journal.pntd.0003873

**Published:** 2015-07-02

**Authors:** Geoffrey K. Isbister, Kalana Maduwage, Ana Saiao, Nicholas A. Buckley, Shaluka F. Jayamanne, Shahmy Seyed, Fahim Mohamed, Umesh Chathuranga, Alexandre Mendes, Chandana Abeysinghe, Harindra Karunathilake, Indika Gawarammana, David G. Lalloo, H. Janaka de Silva

**Affiliations:** 1 Clinical Toxicology Research Group, University of Newcastle, Newcastle, New South Wales, Australia; 2 South Asian Clinical Toxicology Research Collaboration (SACTRC), Faculty of Medicine, University of Peradeniya, Peradeniya, Sri Lanka; 3 Clinical Pharmacology, Sydney Medical School, University of Sydney, Sydney, New South Wales, Australia; 4 Department of Medicine, Faculty of Medicine, University of Kelaniya, Ragama, Sri Lanka; 5 School of Electrical Engineering and Computer Science, University of Newcastle, Newcastle, New South Wales, Australia; 6 District Hospital Hingurakgoda, Hingurakgoda, Sri Lanka; 7 General Hospital Matara, Matara, Sri Lanka; 8 Clinical Sciences and International Public Health, Liverpool School of Tropical Medicine, Liverpool, United Kingdom; Universidad de Costa Rica, COSTA RICA

## Abstract

**Background:**

There is limited information on antivenom pharmacokinetics. This study aimed to investigate the pharmacokinetics of an Indian snake antivenom in humans with Russell’s viper bites.

**Methods/Principal Findings:**

Patient data and serial blood samples were collected from patients with Russell’s viper (*Daboia russelii*) envenoming in Sri Lanka. All patients received Indian F(ab’)_2_ snake antivenom manufactured by VINS Bioproducts Ltd. Antivenom concentrations were measured with sandwich enzyme immunoassays. Timed antivenom concentrations were analysed using MONOLIXvs4.2. One, two and three compartment models with zero order input and first order elimination kinetics were assessed. Models were parameterized with clearance(CL), intercompartmental clearance(Q), central compartment volume(V) and peripheral compartment volume(V_P_). Between-subject-variability (BSV) on relative bioavailability (F) was included to account for dose variations. Covariates effects (age, sex, weight, antivenom batch, pre-antivenom concentrations) were explored by visual inspection and in model building. There were 75 patients, median age 57 years (40-70y) and 64 (85%) were male. 411 antivenom concentration data points were analysed. A two compartment model with zero order input, linear elimination kinetics and a combined error model best described the data. Inclusion of BSV on F and weight as a covariate on V improved the model. Inclusion of pre-antivenom concentrations or different batches on BSV of F did not. Final model parameter estimates were CL,0.078 Lh^-1^, V,2.2L, Q,0.178Lh^-1^ and V_P_,8.33L. The median half-life of distribution was 4.6h (10-90%iles:2.6-7.1h) and half-life of elimination, 140h (10^th^-90^th^ percentilesx:95-223h).

**Conclusion:**

Indian F(ab’)_2_ snake antivenom displayed biexponential disposition pharmacokinetics, with a rapid distribution half-life and more prolonged elimination half-life.

## Author Summary

Snake envenoming is a neglected tropical disease that affects hundreds of thousands of people in the rural tropics. Antivenom is the main treatment for snake bites but there is limited information on the pharmacokinetics and appropriate dosing regimen. Most studies have been done in animals and dosing guidelines are based on arbitrary and often irreversible clinical signs. In this study we measured serial antivenom concentrations in patients with Russell’s viper envenoming given antivenom. Using this data we modelled the pharmacokinetics of antivenom in the population and showed that antivenom concentrations had a bi-exponential decay with an initial decrease over 12 hours and then a slow decrease over days. There was significant variability in the dose given which was not affected by the particular antivenom batch given. The presence of venom did not appear to modify the pharmacokinetics of antivenom. Understanding the time course of antivenom in patients with snake envenoming will provide a better basis for antivenom dosing.

## Introduction

Snake envenoming is a major health issue in South and South-eastern Asia [1]. Although antivenom is the most important treatment for snake envenoming, it can cause early systemic hypersensitivity reactions [2, 3], and there is limited evidence to support currently practiced dosing schedules. Dosing and assessment of the effectiveness of antivenom in human envenoming remains controversial and treatment protocols are not based on the kinetics of venom or antivenom. There are few studies of the pharmacokinetics of antivenom, and most of these are in animals [4].

Snake envenoming is a common problem in Sri Lanka and large amounts of antivenom are used throughout the country each year. A number of different Indian antivenoms are currently used and the initial dose ranges from 10 to 20 vials [5–7]. The initial dose is based on ED50 studies and clinical experience by titrating dose against the resolution of coagulopathy and neurotoxicity. However, the clinical effects of envenoming in these species are generally irreversible so determining if enough antivenom has been given and deciding to re-dose is often arbitrary and not based on whether all venom has been bound, or on the pharmacokinetics of antivenom. Measurement of venom and antivenom concentrations in patients with snake bite is required to improve effective initial and repeat dosing [8].

The pharmacokinetics of antivenom are expected to be similar to other intravenous drugs being delivered to the central compartment with zero order input kinetics (constant rate of infusion). Antivenom is then distributed throughout the body and is eliminated by the kidneys and/or the reticuloendothelial system [4]. Decreasing antivenom concentrations in the central compartment are therefore due to both distribution and elimination. Different types of antivenom have different pharmacokinetics due to the difference in their molecular masses [4]. Fab antivenoms have much larger volumes of distribution (V_D_) than F(ab’)_2_ or whole IgG [5, 9]. Most studies of antivenom pharmacokinetics show a biphasic (two-compartment) decline after intravenous administration of whole IgG and F(ab’)_2_ antivenoms, as a result of an initial rapid decline (distribution phase) and a slower decline (terminal elimination phase) [4, 9].

Most studies of the pharmacokinetics of antivenom are in animals [4, 10], and the pharmacokinetics appear to differ between animals making animal models problematic for defining the pharmacokinetics of antivenom in humans [10]. Although there have been several publications of antivenom concentrations in snake envenoming, there are only a few studies of the pharmacokinetics of antivenom in human snake envenoming [4, 5, 9, 11–14]. These studies were all in small numbers of patients using a classic two phase approach, without including input processes (i.e. delivery of the antivenom, usually via an infusion to the central compartment as a zero order process) and providing limited information on the pharmacokinetics and variation between patients.

A population approach to pharmacokinetic analysis is increasingly being used to define the pharmacokinetics of drugs in humans because it provides information about population variability and the need for individualisation of drug treatment. The traditional approach to pharmacokinetic analysis (two stage analysis) estimates the pharmacokinetic parameters for each individual patient and then provides summary statistics which only give a population average and standard error. In contrast the population approach estimates the typical value of each parameter for the population and the variability of the parameters simultaneously. This provides an estimate of unexplained random variation and allows the effects of covariates to be accounted for in the model (e.g. weight, renal function). There are no previously published population pharmacokinetic analyses of antivenom in humans or animals.

The aim of this study was to investigate the pharmacokinetics of antivenom in patients with snake bites using a population based analysis, including an investigation of the covariates that may influence the pharmacokinetics of antivenom.

## Methods

This was a population pharmacokinetic analysis of an F(ab’)_2_ antivenom using data and serial antivenom concentrations collected in snake-bite patients admitted to a single hospital in Sri Lanka. The patients were recruited from within a large cohort of snakebites admitted to the Base Hospital Polonnaruwa in Central Eastern Sri Lanka.

### Ethics statement

The study was approved by the Ethical Review Committee, Faculty of Medicine, University of Peradeniya, Sri Lanka. All patients gave written and informed consent for the collection of clinical data and blood samples.

### Patients

All patients (>15 years old) from October 2010 to March 2012 with a suspected snake bite who presented to the Base Hospital Polonnaruwa were recruited to a prospective cohort study. Those with coagulopathy were then entered in a dose finding randomised clinical trial of fresh frozen plasma. The entry criteria for the trial was a suspected Russell’s viper (*Daboia russelii*) bite with coagulopathy defined as an abnormal 20 minute whole blood clotting test (20WBCT). This resulted in a small number of patients being recruited where Russell’s viper (*D*. *russelii*) venom was not detected and on further testing, hump-nosed viper (*Hypnale* spp.) venom was detected (in some *Hypnale* bites the 20WBCT and coagulation studies may be abnormal [15, 16]).

In this pharmacokinetic study, patients were only recruited from the clinical trial and were included if they had serial serum collection for antivenom measurement and complete demographic details (including weight). All patients received the Indian polyvalent snake antivenom intravenously manufactured by VINS Bioproducts Limited (batch numbers: 1060 [MFD 2008], 1096 [MFD 2009], 1102 [MFD 2009], 01015/10-11 [MFD 2010], 01AS11112 [MFD 2011]). For a dose of antivenom, each of 10 vials of antivenom are reconstituted in 10ml of normal saline for a total of 100ml of antivenom. From a 500ml bag of normal saline 100ml volume is removed and replaced by the 100ml of antivenom so the 10 vials are administered in a total of 500ml of normal saline. This is given over 1 hour.

### Data collection

The following data were collected prospectively in all cases: demographics (age, sex and weight), time of the snake bite, clinical effects (local envenoming, coagulopathy, bleeding and neurotoxicity) and antivenom treatment (dose, time of administration and antivenom batch number). Blood samples were collected for research on admission and regularly throughout each patient admission. Blood was collected in serum tubes for venom-specific enzyme immunoassay (EIA) and antivenom EIA. All blood samples were immediately centrifuged, and then the serum aliquoted and frozen initially at -20°C, and then transferred to -80°C within 2 weeks of collection.

### Enzyme immunoassays for venom and antivenom

A sandwich enzyme immunoassay was used to measure antivenom in serum samples as previously described [8, 17]. The plate was first coated with Russell’s viper venom and then stored and blocked overnight. Serum was then added to the plates. The detecting antibodies were conjugated with horseradish peroxidase. Russell’s viper (*D*. *russelii*) and hump-nosed (*Hypnale* spp.) viper venoms were measured in samples with a venom specific enzyme immunoassay as previously described [6, 8, 17]. Briefly, polyclonal IgG antibodies were raised in rabbits against Russell’s viper (*D*. *russelii*) and hump-nosed viper (*Hypnale* spp.) venom. The antibodies were then bound to microplates and also conjugated to biotin for a sandwich enzyme immunoassay using streptavidin-horseradish peroxidase as the detecting agent. All samples were measured in triplicate, and the averaged absorbance converted to a concentration using a standard curve made up with serial dilutions of antivenom and using a sigmoidal curve. The limit of quantification for the antivenom enzyme immunoassay assay was 40μg/ml and for the venom enzyme immunoassay was 2ng/mL for Russell’s viper and 0.2ng/ml for hump-nosed viper.

### Pharmacokinetic analysis

Patient data was analysed using MONOLIX version 4.2 (Lixoft,Orsay, France. www.lixoft.com). MONOLIX uses the Stochastic Approximation Expectation Maximization algorithm (SAEM) and a Markov chain Monte-Carlo (MCMC) procedure for computing the maximum likelihood estimates of the population means and between-subject variances for all parameters [18]. One, two and three compartment models with zero order input and first order elimination kinetics were assessed and compared to determine the best structural model. Proportional and combined models were evaluated for the residual unexplained variability. Method M3 was used to deal with antivenom concentrations below the limit of quantification (BLQ) [19]. Between-subject variability (BSV) was included in the model and assumed to have log-normal distribution.

Models were parameterized in terms of volume of distribution (V_D_; V, V_P_, V_P2_), clearance (CL), inter-compartmental clearance (Q; Q1, Q2) and relative bioavailability (F) for either 1-, 2- or 3-compartment models. Initial estimates of parameters were taken from a previous pharmacokinetic study of anti-venom [9].

Uncertainty in antivenom dose was included in the model by allowing BSV on F to account for batch to batch variation in antivenom (five different batches) and for variation within batches. F was fixed to 1 and the BSV was estimated for each patient similar to including uncertainty on dose as previously described [18]. The BSV on F was plotted for each batch to determine if there was a difference between batches.

The effect of covariates, including age, sex, weight, and pre-antivenom concentrations in patients with detectable venom, were explored by visual inspection of the individual parameter estimates versus the covariate of interest. Age, sex and pre-antivenom concentrations were not included in the final model evaluation due to the absence of an association visually. The influence of weight (wt) on volume was included in the modelling process. Weight was assumed to be related to V by a power function. The covariate was centred to the average weight. Thus in the model the estimation of the effect of weight on volume is:

$${\text{V = θ}}_{\text{V}}{\text{ x (wt/wt}}_{\text{av}}\text{)}\wedge {\text{f}}_{\text{wt}}$$

Where θ_V_ is the typical value of volume of distribution, wt is the individual patient weight, wt_av_ is the average weight and f_wt_ accounts for the influence of wt on volume.

Model selection decisions were based on a decrease in the objective function (OFV), a decrease in residual error, clinical relevance of the pharmacokinetic parameters and goodness of fit plots. The log likelihood was computed for each model and used to discriminate through the difference in log likelihood (−2LL). A p-value of 0.05 was considered statistically significant, equivalent to a drop in OFV by 3.84.

### Simulations

From the final model we simulated 1000 patients using the individual predicted patient parameters from the final model with MatLab to explore different initial doses and repeat doses. The following scenarios were explored:

1. One dose (10 vials) of antivenom given with infusions rates of 20 minutes, 1 hour and 2 hours.
2. Two doses of antivenom given, each over 1 hour and 6 hours apart.
3. Two doses of antivenom given, each over 1 hour and 12 hours apart.

The median antivenom concentration versus time was plotted with 10% and 90% percentiles.

## Results

### Patients

There were 75 patients with a median age of 38 years (16 to 64y) and 64 were male. Seventy one were Russell’s viper envenoming cases and 52 of these had detectable venom prior to the administration of antivenom. Four patients had hump-nosed viper envenoming (confirmed by detectable hump-nosed viper venom). In all four patients with hump-nosed viper envenoming there was a steady decline of venom concentrations despite the administration of antivenom consistent with the antivenom not being raised against this snake venom. In nineteen patients meeting the inclusion criteria venom was not detected prior to antivenom, most likely because the blood was collected prior to envenoming. The demographics of the patients are listed in Table 1.

**Table 1:** Demographics and clinical information of 75 patients who were administered Indian antivenom, including clinical features of envenoming, treatment and outcomes.

	Median (range)	Number (%)
**Age (years)**	38 (16 to 64)	75 (100)
**Male**	-	64 (85)
**Weight (kg)**	57 (40 to 70)	
**Clinical Effects**		
Local envenoming		73 (97)
Coagulopathy (20WBCT+)		75 (100)
Systemic Bleeding		26 (35)
Neurotoxicity (ptosis)		32 (43)
**Antivenom batch**		
1060 (MFD 2008)		1 (1)
1096 (MFD 2009)		8 (11)
1102 (MFD 2009)		12 (16)
01015/10-11 (MFD 2010)		40 (53)
01AS11112 (MFD 2011)		14 (19)
**Antivenom dose (vials)**	18 (8 to 40)	
**Repeat antivenom dose**		21 (28)
**Median pre antivenom venom concentration ng/ml (range)**	169 (2 to 2805)^†^	52 (69)
**Fresh Frozen Plasma (FFP)**		
2 Units FFP		44 (59)
4 Units FFP		14 (19)
8 Units FFP		1 (1)
**Length of hospital stay (days)**	2 (1 to 10)	
**Antivenom concentrations (μg/ml)**	1916 (27 to 13673)	392 samples*

* Samples with antivenom measured in them from a total of 510 samples, 128 had no detectable antivenom;

^†^ Russell’s viper venom only detectable in 53 patients; 20WBCT– 20 minute whole blood clotting test.

**Fig 1:**
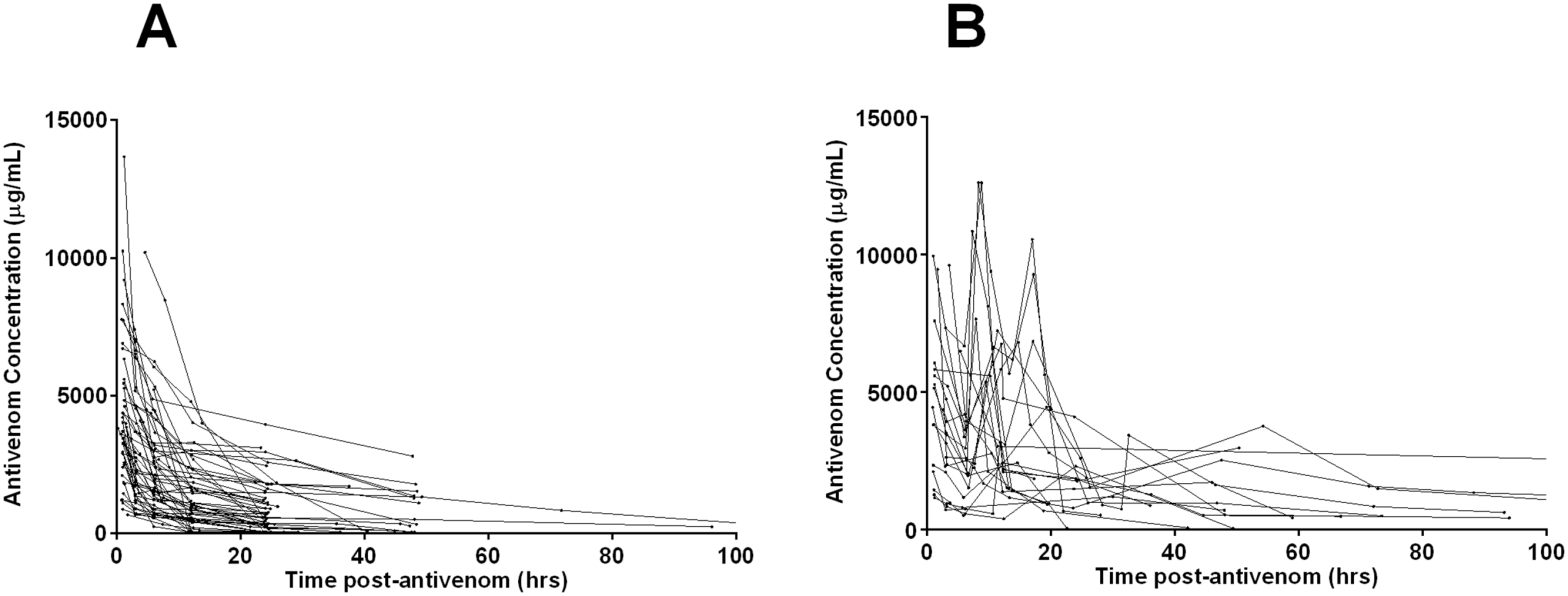
Plots of the observed antivenom concentration (μg/ml) versus time for patients given a single dose of antivenom (A), and for patients given multiple antivenom doses (B).

There were 510 antivenom concentration data points but only 411 had detectable antivenom, the other 99 were serial samples after the disappearance of antivenom. There were 54 patients who had a single dose of antivenom who had 265 antivenom concentration measurements with a median of five antivenom concentrations in each patient (Range: 2 to 10), and a median antivenom concentration of 1607μg/ml (Range: 40 to 13673μg/ml). There were 21 patients who had multiple doses of antivenom who had 146 antivenom concentrations with a median of seven antivenom concentrations in each patient (Range: 3 to 11) and a median antivenom concentration of 2293μg/ml (Range: 40 to 12599μg/ml). The observed concentration versus time data is shown in Fig 1.

### Pharmacokinetic analysis

A two compartment model with zero order absorption and linear elimination kinetics and a combined error model best described the data. The final model incorporated BSV on F, which was fixed to 1 to allow variability between patients in dose. The model also incorporated weight as a covariate with a power effect on central volume, V. The inclusion of pre-antivenom concentrations on BSV of F did not improve the model. Plots of the BSV on F versus the batch number showed no relationship between the batch and BSV on F (S1 Fig). The final model parameter estimates were CL, 0.078 Lh^-1^, V, 2.2L, Q, 0.178Lh^-1^ and V_P_, 8.33L. The median half-life of distribution was 4.6h (10^th^-90^th^ percentiles: 2.6 to 7.1h) and the half-life of elimination, 140h (10^th^-90^th^ percentiles: 95 to 223h). There was no difference in the parameter estimates between those with Russell’s viper envenoming with detectable venom prior to antivenom (52), those with Russell’s viper envenoming and no detectable venom prior to antivenom (19) and those with hump-nosed viper envenoming (S2 Fig). S3 and S4 Figs shows the goodness-of-fit plots for the final model. The individual PK parameter estimates from the base models with modelling decisions and final model parameters are described in Table 2. There was also no difference in parameter estimates between patients given 1 dose of antivenom and those given 2 doses, or between patients with different initial venom concentrations (S5 Fig).

**Table 2:** Parameter estimates using Monolix version 4.2.

	Base model	Model 1	Model 2	Model 3 (Final)
Mean value (rse%)		Including F	Including weight on V	Including F and weight on V
**Structural model (θ)**				
CL (Lh^-1^)	0.0445 (50)	0.038 (65)	0.129 (23)	0.0779 (34)
V (L)	2.23 (9)	1.98 (11)	2.12 (11)	2.16 (10)
Q (Lh^-1^)	0.171 (21)	0.271 (20)	0.107 (38)	0.178 (31)
Vp (L)	17.6 (50)	14.1 (43)	3.27 (121)	8.33 (52)
*f*_wt_	-	-	0.137 (96)	0.132 (84)
F	-	1 (-)	-	1 (-)
**Between subject variance (ω)**				
Cl	1.64 (47)	0.451 (81)	0.886 (30)	0.715 (46)
V	0.42 (22)	0.0627 (74)	0.419 (30)	0.188 (126)
Q	0.738 (33)	0.237 (71)	2.57 (84)	0.533 (57)
Vp	1.24 (57)	1.62 (56)	0.782 (282)	0.836 (125)
F	-	0.309 (23)	-	0.197 (42)
**Objective Function (*-2 log-likelihood value)***	6840.81	6823.54	6835.96	6820.75

CL = clearance, V = volume of the central compartment, Q = intercompartmental clearance, Vp = volume of the peripheral compartment, fwt = effect of weight on V, F = relative bioavailability.

**Fig 2:**
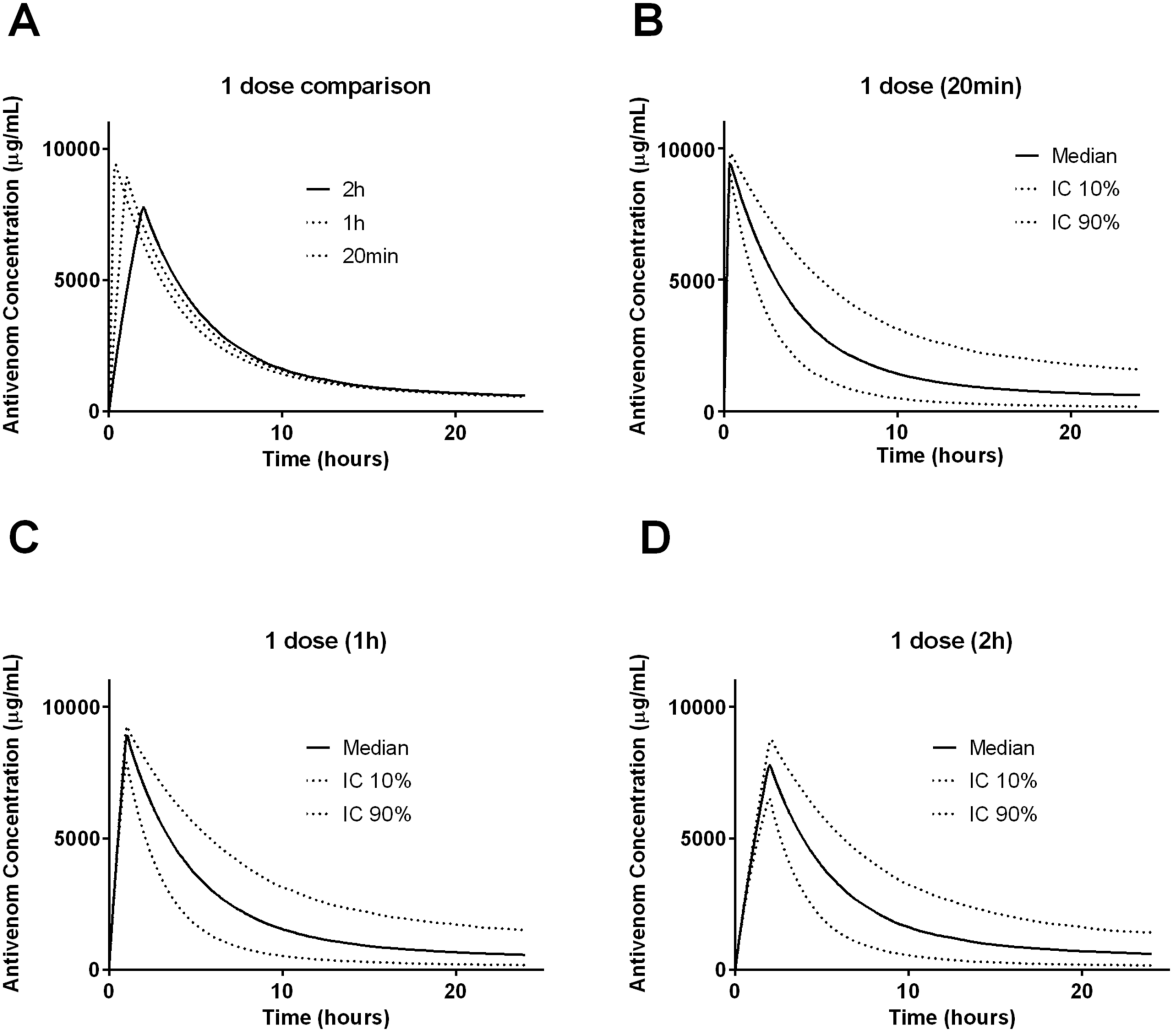
Plots of antivenom concentration versus time for 1000 patients simulated from the final model individual predicted patient parameters for 10 vials of antivenom given over 20min, 1h and 2h, comparing the median concentrations for all three regimens (A), and the median and 10% and 90% percentile concentrations for a 20 minute infusion (B), 1 hour infusion (C) and 2 hour infusion (D).

**Fig 3:**
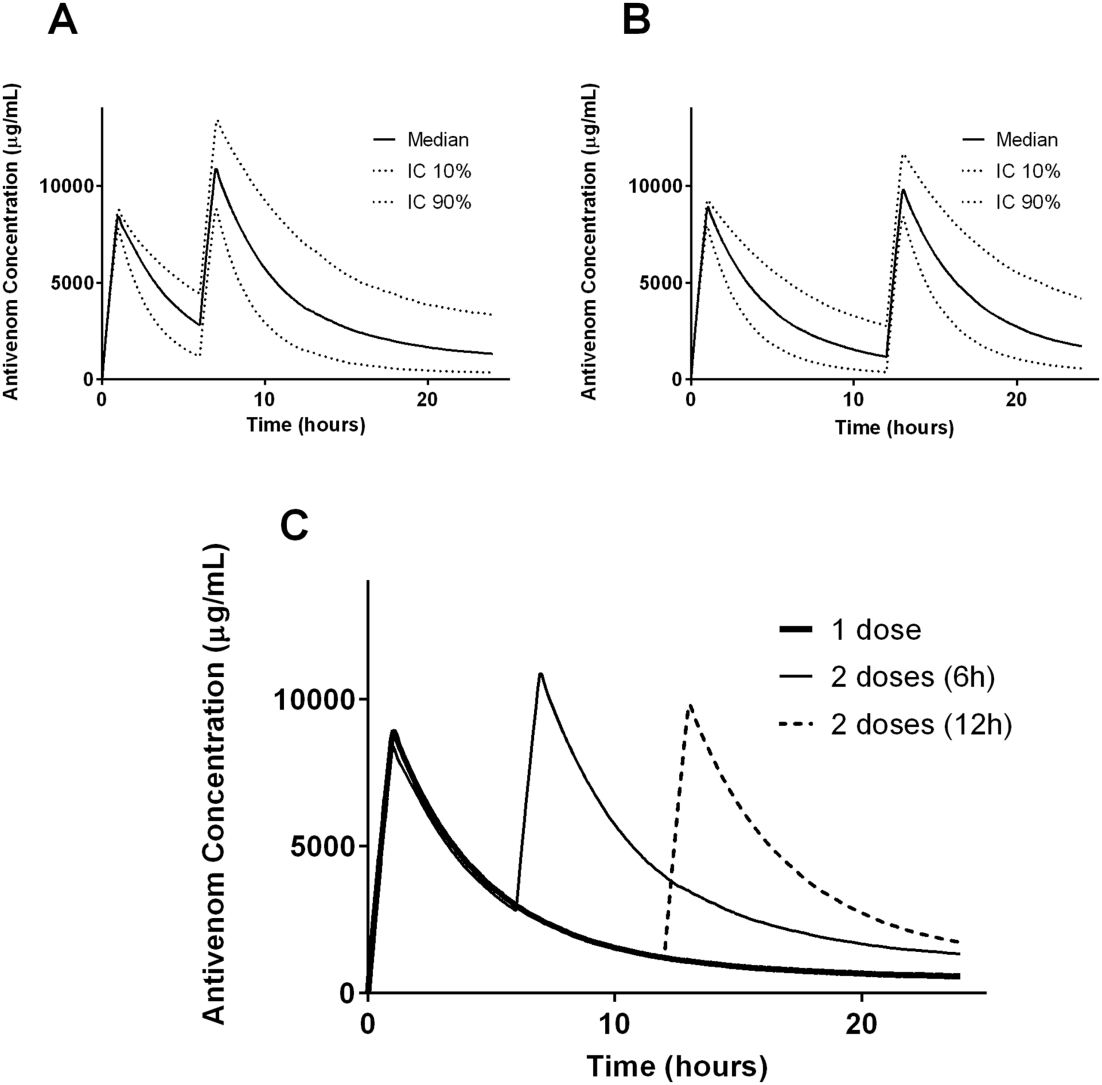
Plots of antivenom concentration versus time for 1000 patients simulated from the final model individual predicted patient parameters for patients given two doses of antivenom, the first given 10 vials over 1 hour and then repeated at 6 hours (A) and the second given 10 vials over 1 hour and then repeated at 12 hours (B), showing median, 10% and 90% percentile concentrations. The two regimens with repeat doses are compared to a single dose in Panel C.

Simulations for one dose (10 vials) of antivenom given over 20 minutes, 1 hour and 2 hours shows there is a slightly lower and later peak antivenom concentration with slower infusions (Fig 2). Simulations for two doses of antivenom shows that antivenom concentrations decrease rapidly after each dose and there are low but persistent levels of antivenom after one dose and both two doses regimens (Fig 3).

## Discussion

The study adds to the limited information available on the pharmacokinetics of antivenom in humans supporting previous studies [4, 9]. Indian F(ab’)_2_ snake antivenom displayed biexponential disposition pharmacokinetics, with a rapid half-life of distribution and a much longer half-life of elimination. Weight accounted for some of the variability in the central volume, and the volumes of the central and peripheral compartment were consistent with a large molecule which does not have a large volume of distribution. Including variability on F improved the model showing that there was significant random variability in dose. The plots in Figs 2 and 3 show the expected antivenom concentration profiles in the first 24 hours after administration.

Previous human and most animal studies have also shown a biexponential decay in antivenom concentrations [4, 9, 20, 21], with similar values for the distribution half-life of 2 to 4 hours and much longer elimination half-life of 90 to 230h. Previous studies have been small with 10 or less patients in each analysis (for different antivenoms) and a classic two phase approach has been undertaken. Such an approach will over-estimate the error and not account for true random variability or covariate effects. In this study we have undertaken a population approach, which provides information on the variability of the pharmacokinetics in the population and an improved model by including weight and variability in dose. Previous studies have not shown why they chose particular models (2-compartment versus 3-compartment), with no statistical criteria or goodness of fit plots.

Some previous animal models and one human study have described the pharmacokinetics with a tri-exponential decay in animals [14, 22, 23]. These analyses have not included an input process in the analysis which will bias the estimation of the disposition parameters, particularly with three or more compartments when the initial very short half-life is similar to the time of the input phase. Ismail et al. estimated the initial rapid half-life in animals to be 0.2h and Vazquez et al estimated it to be 0.25h, which are both similar to the usual infusion rate of antivenom over 10 to 30 minutes. It is possible that there is only 2-compartmental disposition kinetics in these studies, and future pharmacokinetic analyses need to include an input phase in the model. A possible limitation of our study was that there may have been insufficient sampling in the initial period after antivenom administration to detect a third compartment. In contrast to this, Vazquez et al were likely to have taken samples in the input phase, since the first sample was taken 5min after antivenom administration, although they do not report the infusion time or rate [14].

One animal study of a F(ab’)_2_ has shown that the pharmacokinetics of antivenom are the same in envenomed and non-envenomed rabbits [24]. This is consistent with this study demonstrating that pre-antivenom venom concentrations did not influence the pharmacokinetics of antivenom, including different initial venom concentrations (S5 Fig). However, this may be different for Fab antivenoms where high molecular weight toxins may change the route of elimination from renal (for free Fab antivenom) to phagocytosis/reticulo-endothelial system for Fab-toxin molecules. The latter has been shown in rabbits with anti-*Vipera* Fab antivenom [25].

There has always been concerns about the variability between different batches of antivenom leading to potential differences in the dose administered between batches. The study did not support this concern and found that there was no difference in F on average between different batches (S1 Fig). However, the study found that including between subject variability on relative bioavailability did improve the model. This suggests there was random variability in the dose administered which is likely to be due to variable losses occurring during reconstitution of the individual freeze dried vials of antivenom. So, although there may be variability between batches, the variability in dosing errors appears to be larger than the differences between batches.

There are a number of limitations to the study including the fact that the sample collection was not optimally designed and sample times (windows) were based on timing of clinical samples and other research assays required for the clinical trial. This is unlikely to have a major influence on the analysis because a population approach will allow for both sparse and rich sampling in patients. Another issue is that antivenom is not a pure substance and consists of varying amounts of polyclonal antibodies to multiple toxins in the venom with varying affinities. However, the assay uses a single detecting antibody (anti-horse antibody), so will detect all antibodies against the snake toxins irrespective of their toxin target or affinity. Finally, the assay will only detect antibodies that bind to the snake toxins. In most antivenoms, specific antibodies to snake toxins make up only 10 to 20% of the total protein/immunoglobulin content. This is unlikely to have affected the pharmacokinetic analysis because only immunoglobulins binding to snake toxins are relevant to the analysis.

This population pharmacokinetic analysis demonstrates that Indian F(ab’)_2_ antivenom has biexponential disposition kinetics and following an initial decline in antivenom concentrations in the first 12 hours, low concentrations are present for days after administration. The study demonstrates that the antivenom concentrations were not affected by the initial venom concentrations suggesting that sufficient antivenom in excess of the venom was being administered. Understanding the pharmacokinetics of antivenom may assist in improving antivenom dosing by matching antivenom pharmacokinetics to the neutralisation of venom (pharmacodynamics), as well as clinical effects.

## Supporting Information

S1 Fig Plots of the relative fraction absorbed (F) versus batch number. (TIF)

S2 Fig Plots comparing Russell’s viper envenomed patients to hump-nosed viper envenomed patients for clearance (A), central volume (B), inter-compartmental clearance (C), peripheral volume (D), half-life of distribution (E), half-life of elimination (F) and the relative fraction absorbed (G). (TIF)

S3 Fig Goodness-of-fit plots including a visual predictive check (VPC) showing the observed data with the 10th, 50th, and 90th percentiles. (TIF)

S4 Fig A plot of observations versus individual predictions from the final model. (TIF)

S5 Fig Plots comparing patients receiving one versus two doses of antivenom and comparing 3 different pre-antivenom venom concentrations (Low– 2 to 60ng/ml; Medium– 61 to 300ng/ml; High—>300ng/ml) for clearance (A), central volume (B), inter-compartmental clearance (C), peripheral volume (D), half-life of distribution (E), half-life of elimination (F) and the relative fraction absorbed (G). (TIF)
