# Supplementary figures and images for: Population Pharmacokinetics of an Indian F(ab')2 Snake Antivenom in Patients with Russell's Viper (Daboia russelii) Bites
Source: PLoS Negl Trop Dis. 2015 Jul 2;9(7):e0003873. doi: 10.1371/journal.pntd.0003873 (PMC4489840; doi:10.1371/journal.pntd.0003873)

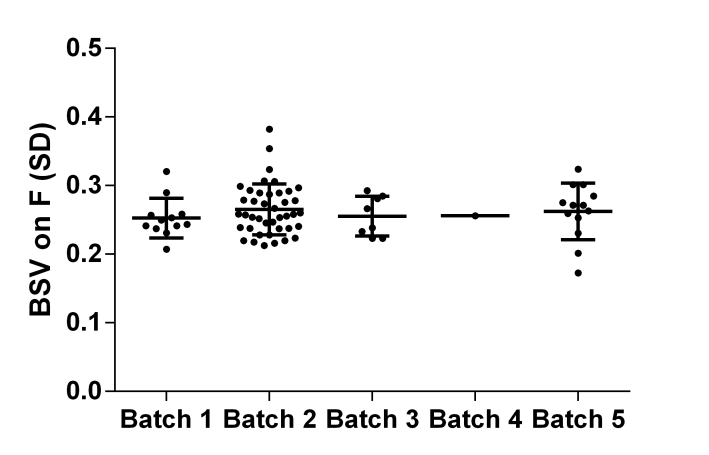

Supplement: S1 Fig — (TIF) [file pntd.0003873.s001.tif]

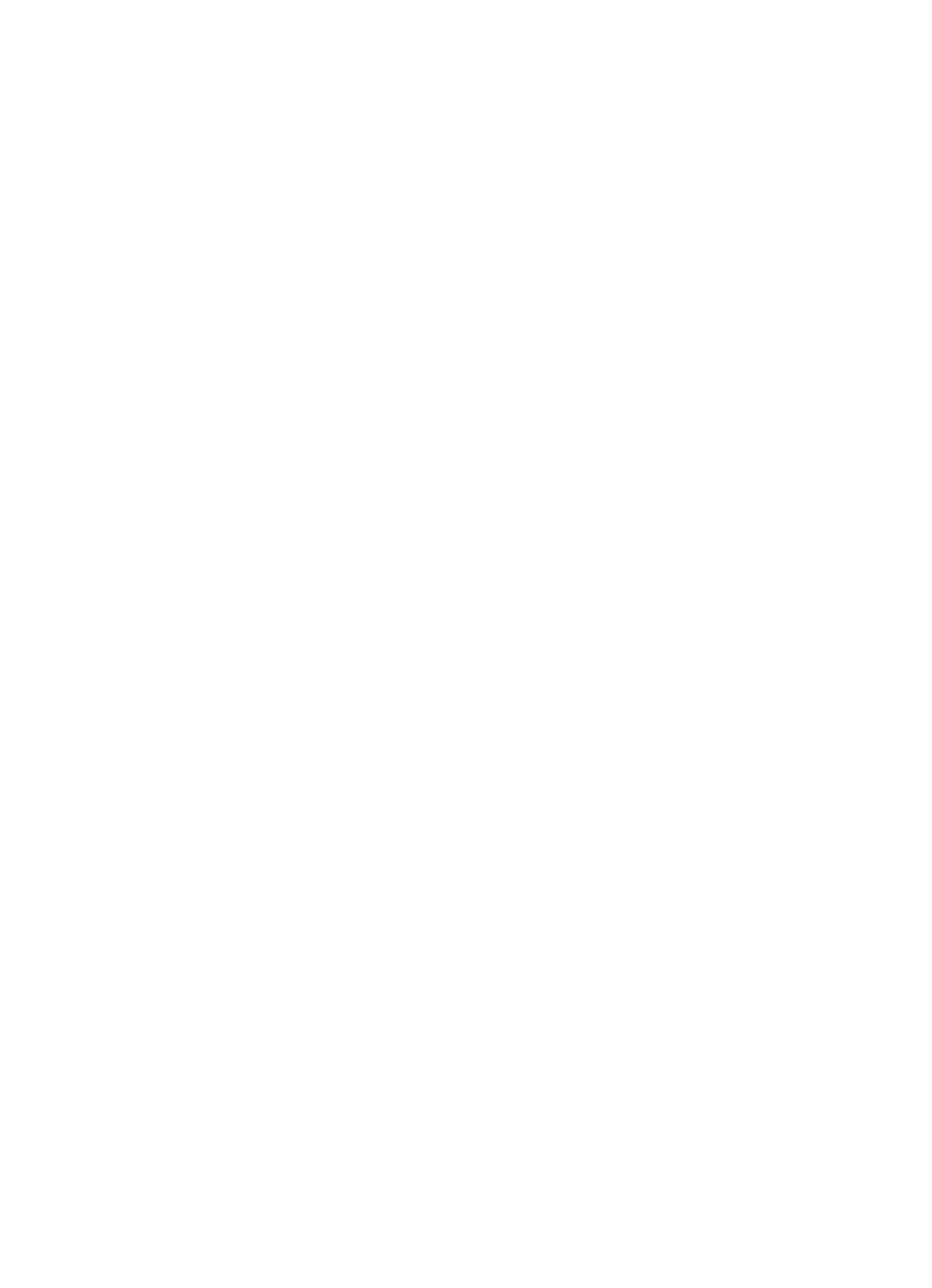

Supplement: S2 Fig — (TIF) [file pntd.0003873.s002.tif]

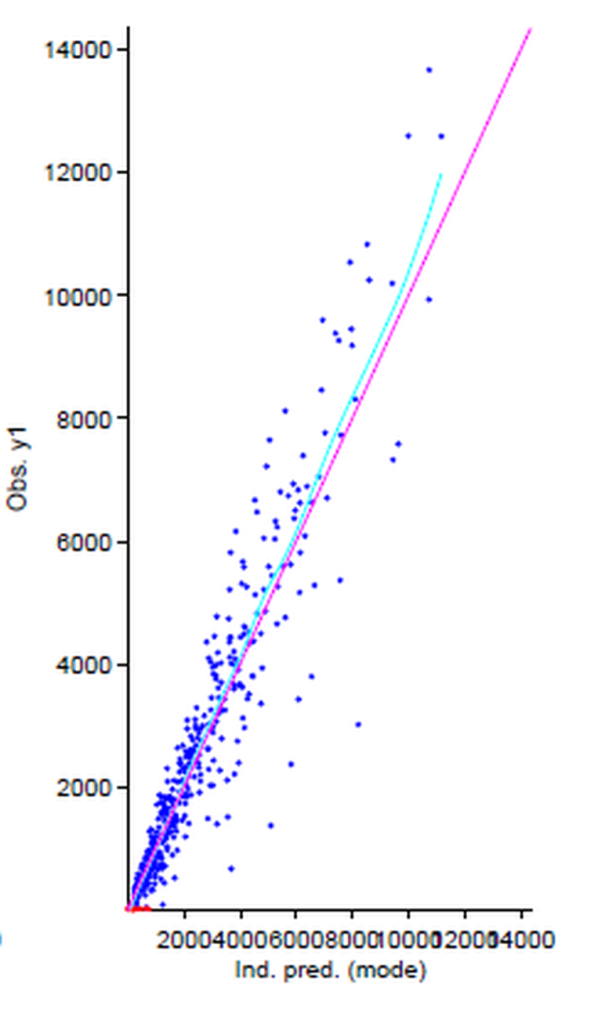

Supplement: S3 Fig — (TIF) [file pntd.0003873.s003.tif]

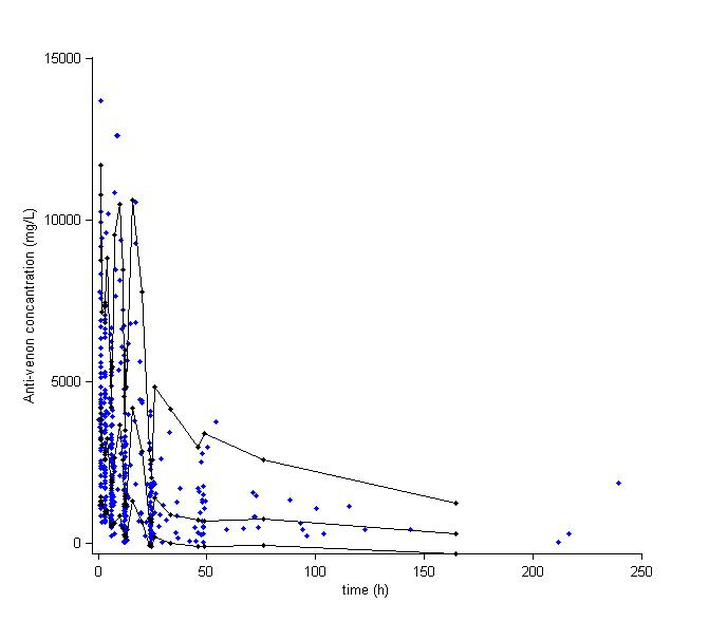

Supplement: S4 Fig — (TIF) [file pntd.0003873.s004.tif]

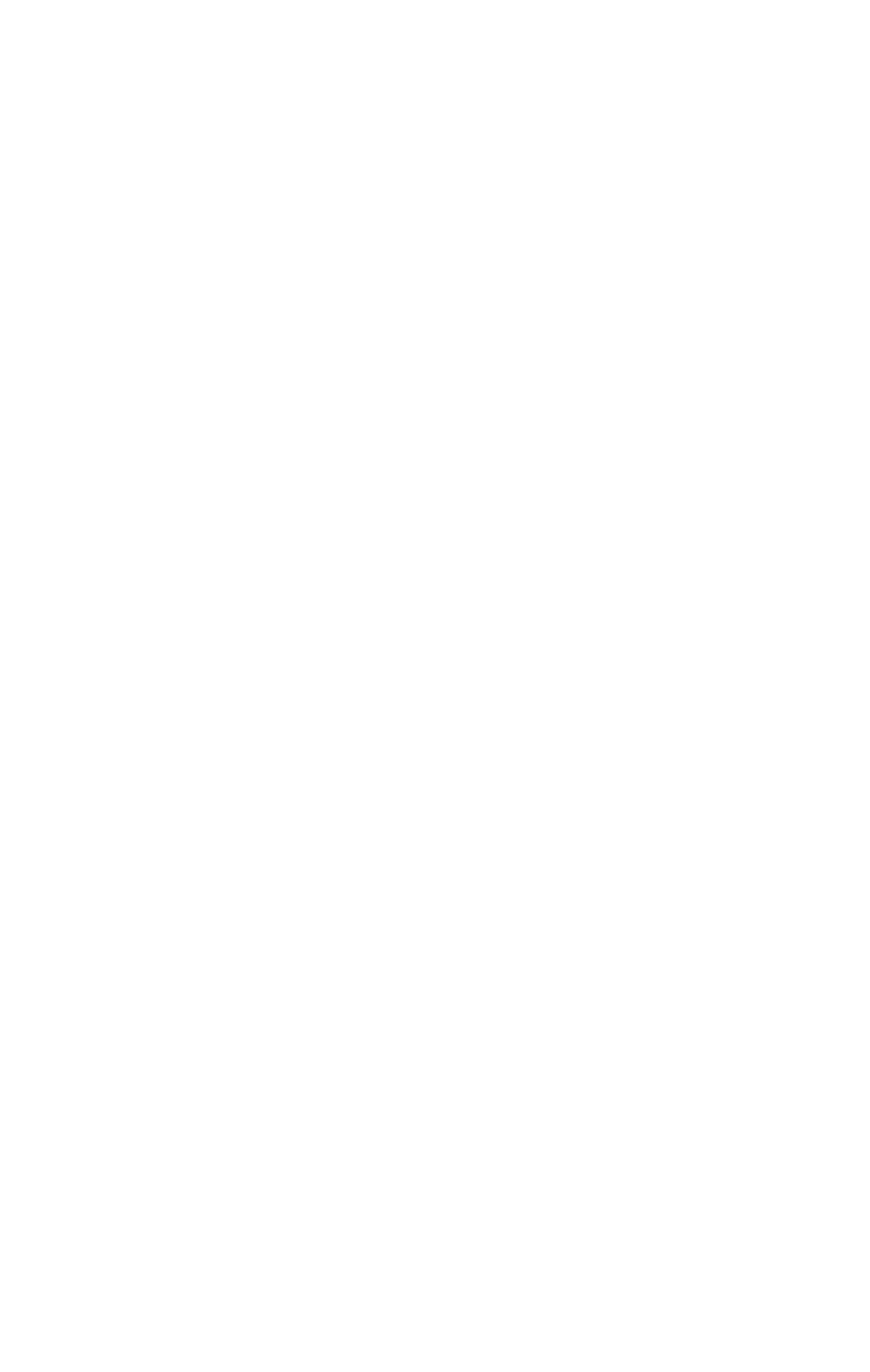

Supplement: S5 Fig — (TIF) [file pntd.0003873.s005.tif]
